# K-nearest-neighbor algorithm to predict the survival time and classification of various stages of oral cancer: a machine learning approach

**DOI:** 10.12688/f1000research.75469.1

**Published:** 2022-01-20

**Authors:** Rashmi Siddalingappa, Sekar Kanagaraj

**Affiliations:** 1 Computational and Data Sciences, Indian Institute of Science, Bangalore, Karnataka, 560012, India

**Keywords:** Cross-Validation, classification, Electronic Medical Records (EMR), K-nearest neighbor (KNN), Regression

## Abstract

**Background:**For years now, cancer treatments have entailed tried-and-true methods. Yet, oncologists and clinicians recommend a series of surgeries, chemotherapy, and radiation therapy. Yet, even amidst these treatments, the number of deaths due to cancer increases at an alarming rate. The prognosis of cancer patients is influenced by mutations, age, and various cancer stages. However, the association between these variables is unclear.

**Methods:** The present work adopts a machine learning technique—k-nearest neighbor; for both regression and classification tasks, regression for predicting the survival time of oral cancer patients, and classification for classifying the patients into one of the predefined oral cancer stages. Two cross-validation approaches—hold-out and k-fold methods—have been used to examine the prediction results.

**Results:** The experimental results show that the k-fold method performs better than the hold-out method, providing the least mean absolute error score of 0.015. Additionally, the model classifies patients into a valid group. Of the 429 records, 97 (out of 106), 99 (out of 119), 95 (out of 113), and 77 (out of 91) were classified to its correct label as stages – 1, 2, 3, and 4. The accuracy, recall, precision, and F-measure for each classification group obtained are 0.84, 0.85, 0.85, and 0.84.

**Conclusions:** The study showed that aged patients with a higher number of mutations than young patients have a higher risk of short survival. Senior patients with a more significant number of mutations have an increased risk of getting into the last cancer stage

## Introduction

In India, nearly 1300 people die every day due to cancer, as per the Indian Council of Medical Research (ICMR) reports. The cancer rate is doubled and is likely to increase in the upcoming years ( Takiar Ramnath, 2010). On the contrary, cancer treatments are considerably improved over the past two decades. Structurally, cancer is caused by several mutations and the associated genes ( Sanchez-Vega *et al.*, 2018). The human body undergoes several mutations, but not all lead to cancer. When the gene is mutated, the activities it performs on the cell take a toll, disrupting the cell’s behavior. They may further turn cancerous. The information regarding cancer stages— *de-novo* or metastatic stage, diagnostic process, and survival time are obtained by studying and understanding cancer genes. Fascinatingly, different fields are coming together to develop strategies and techniques extensively applied to treat cancer patients. Nonetheless, the death rate due to cancer is still increasing. Earlier studies indicate that a cancer patient’s survival time is directly proportional to age, mutated genes, and the number of mutations ( Smith, Joan. C, 2018). Thus, the survival prediction at an early stage is helpful in many ways, such as; 1) the surgical intervention could be reduced, 2) treatment could be altered based on body mass index and nutritional screening ( Cavagnari, 2019) to avoid rapid cell proliferation caused by nutrient deprivation, 3) medication and therapies targeting proteins and the related mutations could be introduced, 4) the survival-predicting biomarkers responsible for oral cancer could be learned, and 5) mutation-driven drug discoveries could be made. Further, these predictions and classifications could help clinicians and oncologists determine the patients’ mortality rate and alter the prognosis procedure to better deal with cancer patients. However, the task of predicting survival time is difficult as “the cleaned” and adequately curated data are not available in medical repositories, which come with other complexities with data handling challenges such as data format, dimension scalability, data security, and privacy. Machine learning (ML) could be a one-stop solution to all these limitations. Machine learning techniques require little or no human intervention for processing, decision making, and building models based on system inputs. Several correlations exist between machine learning and medical fields, based on which tremendous results have been achieved ( Huang *et al.*, 2020a). Research programs are being implemented in multinational companies such as Google ( Liu, 2020), IBM ( Matteo Manica, 2019), and Microsoft ( Oktay, 2020) to expand a horizon for new ideas in both ML and medical diagnosis. The key processes in the medical world are information retrieval, data analysis, mining patterns from these data, and eventually extracting features that could help clinicians during treatment. Combining the fundamentals of machine learning and advances achieved in the medical field in cancer treatment could be immensely beneficial. To this end, in the present study, a machine learning technique—k-nearest neighbor (KNN) algorithm—is applied to provide significant insights into the relationship between clinical factors such as age, mutated genes, and mutations and its impact on the survival time of the cancer patients. To the best of the author’s knowledge, no other research studies have considered these factors to analyze their effect on survival time. Further, the patients’ medical record is classified into one of the various oral cancer stages. The research study aims to; i) identify the contributing clinical factors for survival time of oral cancer patients, ii) model an ML-based survival time predictor to generate a clinical report on the go, anywhere and anytime accessible, iii) classify patients into a different stage of oral cancer based on the prediction results, iv) understand the relationship between prognostic markers for survival time and stages of oral cancer, iv) validate the results using MAE and F-scores.

The remainder of this paper is organized as; section 2 discusses the research background in connection with clinical factors and survival time. Section 3 discusses the materials and methods adopted in the present research study and the KNN algorithm used for classification and regression tasks. Section 4 demonstrates the experimental analysis and results for the proposed approach. Section 5 illustrates the validation of the results used to evaluate the system’s performance. Section 6 elaborates on the shortcomings of the proposed work along with the scope for future study. Section 7 concludes the research paper.

### Research background

Several studies on oral cancer indicate that it is one of the most prevalent cancers worldwide ( Cervino Gabriele, 2019). Tackling the mortality rate is crucial as the percentage of people diagnosed with oral cancer is considerably growing. According to the GLOBOCAN 2008 estimate ( Ferlay *et al.*, 2010), 7.6 million deaths have already occurred owing to oral cancer; further, in India, approximately 20 per 100,000 are diagnosed with oral cancer every day, of which 64.8% are males and 35.2% females. In the US, as per the American Cancer Society’s (ACN) Cancer Facts and Figures 2020 ( Siegel, Rebecca L,2020), at least one person is killed every hour because of oral cancer, and approximately 38,330 men and 14,880 women are being diagnosed with oral cancer every year. The estimate of oral cancer deaths in China is approximately 0.89/100,000, and hospitals encounter roughly around 52,500 new cases every year ( Zhang, Shao Kai, 2015). In Saudi Arabia, the annual incidence is evaluated with >3.29% cases ( Al-Jaber, 2016). These statistics demonstrate the necessity to improve oral cancer treatments. Han-Jun Cho *et al.* used machine learning techniques to draw the association between specific gene mutations and the survival factor in lung squamous cell carcinoma ( Cho, Han Jun 2018); to this end, the RapidMinor tool was adopted for the implementation, and feature extraction was realized using the Chi-Squared test and correlation algorithms. Further, they used various classification algorithms such as Naïve Bayes, KNN, support vector machine (SVM), and decision trees to yield specific gene mutations efficiently; Fisher’s extract test and Kaplan–Meier analysis were adopted for the data analysis. The work is implemented on the cancer genome atlas (TCGA) lung adenocarcinoma (LUAD) with the clinical information of 471 patients. Matlak and Szczurek ( 2017) showed the type of mutations that influences the combativeness of cancer and its consequence on the patients’ survival time. The interactions between the mutated genes are accelerated by epistatic communication. The statistical likelihood-ratio test was proposed to recognize the biomarker tumor suppressor p53-binding protein (TP53BP1) that targets poly-ADP (Adenosine diphosphate) ribose polymerase in breast cancer gene-impaired tumors. Zhang *et al.* studied the association between racial disparities related to cancer survival and mutation ( Zhang, Wensheng, 2017). They conducted a consolidative analysis of TCGA clinical samples and genomic data by establishing a relation between racial imbalance and patient survival time. The analyses were based on the Kaplan–Meier standards and achieved an accuracy of 0.69 area under curve (AUC) measure. Chen *et al.* conducted a meta-analysis of the connection between TP53 mutations and survival time in osteosarcoma patients using published data ( Chen, Zhe, 2016). Their study suggested that TP53 mutations had no impact on a patient’s survival time, thus indicating that TP53 mutations could be effectively used as a prognostic marker to estimate patients’ survival rate suffering from osteosarcoma. Their study also demonstrated that altered TP53 had a poor response to chemotherapy and brought down the survival rate of cancer patients. Ling *et al.* constructed a cohort for metastatic breast cancer patients using natural language processing techniques such as semi-supervised classification ( Ling, Albee Y., 2019). They classified the patients’ EMRs into the de-novo stage or recurrent metastatic breast cancer stage, proven with good sensitivity and specificity measures. The SVM technique was used to classify and categorize patients with diabetes based on their EMRs progress notes ( Wright, Adam, 2013). This method achieved an F-score = 0.93 and AUC = 0.956.

## Methods

**Figure 1.**
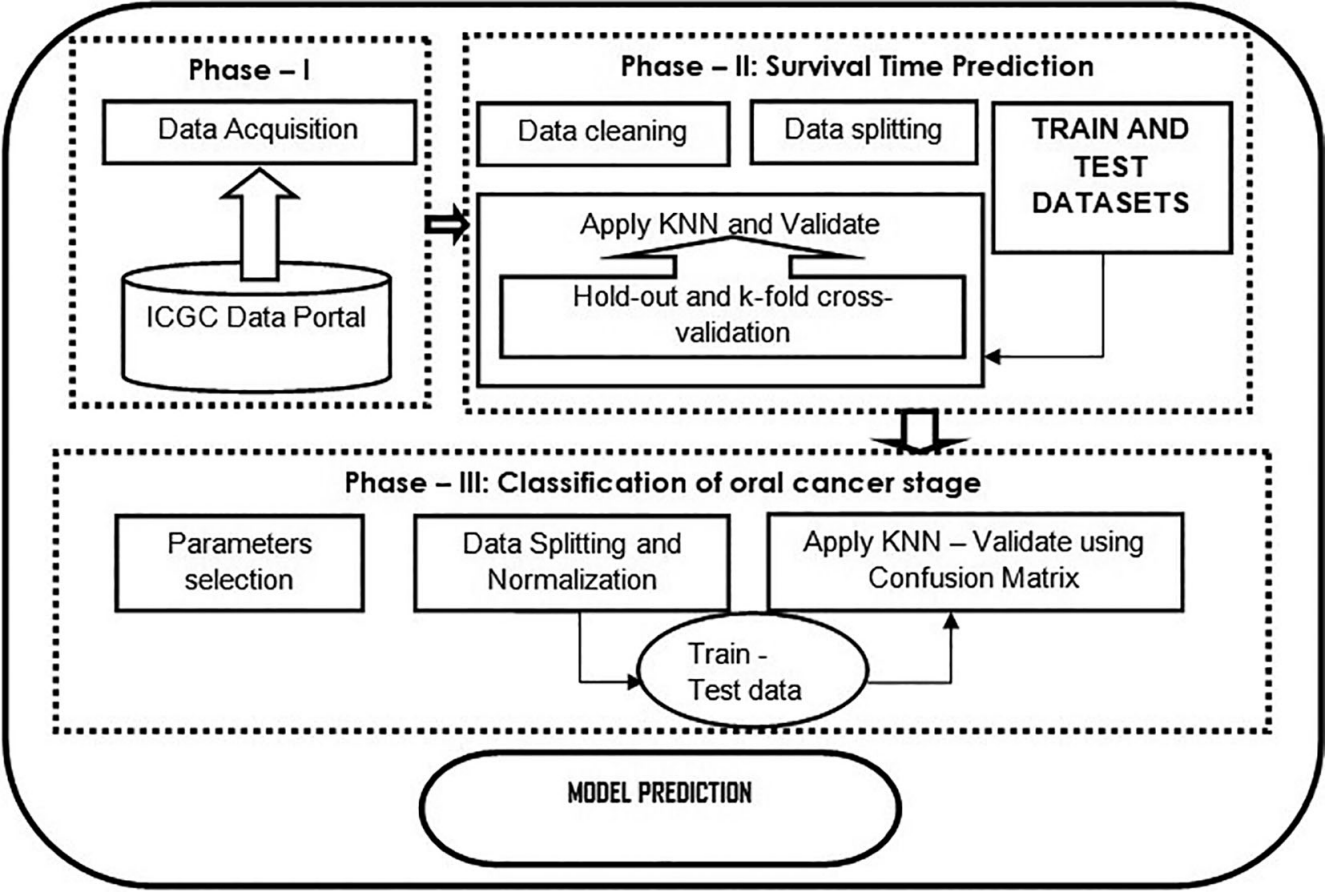
Flowchart of the proposed model described in three phases. Phase – 1 illustrates how data are collected from a data source; phase – 2 shows the data analysis such as data cleaning, data splitting, and applying the KNN algorithm as a regression task to predict the survival time of a cancer patient. Further, validated using standard cross-validation methods; phase – 3 classification using KNN classifier, which is evaluated using the confusion matrix.

The present study aims to predict the number of survival days to understand cancer patients’ mortality rates based on clinical factors and classify them into cancer stages. Figure 1 shows the flowchart of the proposed model.

### Phase – I: Data acquisition

A total of 1,505 oral cancer data records were downloaded from the International Cancer Genome Consortium (ICGC) data portal. The ICGC is a global genomic data-sharing platform that provides the international community with a broad set of genomic data and related cancer types. The dataset contains 50, 178, 243, and 1,034 patients’ records from China, India, Saudi Arabia, and the United States.

### Phase – II: Data analysis

i) Data cleaning: data were extracted in a.tsv file for all four countries. The dataset included several entities; however, not all the fields were required for the implementation. The parameters needed for predicting survival days are age, gender, mutations, and mutated genes. The remaining fields are dropped; ii) Data splitting: Dataset has to be split into training and test set to train an ML model efficiently. However, if the data is inappropriately broken, it may lead to problems such as; 1) overfitting—the model is trained exceptionally well on the training dataset but, the performance is poor on new/test data, and 2) under-fitting—the model does not acquire sufficient information to build the relationship between input datasets, and thus, exhibits a poor performance on training data ( Zhang, Haotian, 2019). Tests were conducted for three categories—70:30, 80:20, and 90:10 to decide on the split ratio. The MAE score was equal to 7.32, 0.012, and 0.089, respectively. A model is considered good if the MAE score is small. Hence, the standard split ratio used in this experimental study is 80:20; iii) normalization: The values of the different fields in the dataset are diverse. For instance, age is denoted by integers and tumor stage by alphanumeric values; further, multiple fields had null or no weights. A standard scale to represent all the fields was required to address this disparity. Normalization was performed before loading the model with the training and test data; iv) implementation platform: The code for the proposed methodologies have been written using python programing (Python 3.9.0) on a Sypder v5.1.1platform *.*

### Phase – III: Survival time prediction and classification of oral cancer into various stages

*i. K-nearest neighbors for regression and classification*

Description: KNN is the most straightforward machine learning algorithm used for both classification and regression tasks. It was initially developed in the early 1950s by Fix and Hodges ( Fix, Evelyn, 1989) from the US Air Force School of Aviation Medicine. Classification involves grouping a given dataset into predefined classes, such as classifying records into one of the four stages of oral cancer ( Chowdhury, Shovan, 2020). In contrast, regression is used for predicting the continuous values such as age, weight, and survival time ( Huang *et al.*, 2020b). The KNN algorithm requires a feature space that contains training data points. This ‘feature similarity’ is used to predict the new data point based on its similarity to the existing data points in the feature space. The algorithm determines the distances between an unknown data point and the nearest ‘k’ training data points and classifies the novel point to that particular class belonging. The value of ‘k’ is based on the number of data points selected from the training dataset. The algorithm primarily starts searching for a numerical feature to best draw the test data point by selecting a metric to calculate the distance ( Gusti Prahmana, 2020). The distances between the new/unknown data point and the ‘k’ points are calculated using a distance measuring metric such as Euclidean distance, Manhattan distance, and Minkowski distance ( Ali, Najat, 2019).

*ii. KNN algorithm for classification and regression task*

#### Algorithm 1: Training KNN algorithm for classification and regression task

Set value of k *(the value of ‘k’ will be set by the user)*

Input: Features expressed by TCGC dataset F={f1,f2,f3, … .fn}, training dataset T={t1,t2,t3, … .tn}, classes C={c1,c2,c3, … .cn}, and the test dataset R

$$\text{Output}:R\in \sum_{i=1}^{n}C$$ (1)

The test data are classified into any one of the classes in ‘C’

**Step: i)** Load the training and test data to the KNN algorithm

**Step: ii)** Set the value of k (the user will set the value of ‘k’)

**Step: iii) For** each test data point, implement the following:

a) Compute the distances from ‘R’ to the nearest points using the Euclidean distance measuring metric.
b) Euclidean distance: Let ‘R’ be the test point, and ‘t _i_’ be the training data point. Therefore, ‘R’ and ‘t _i_’ are vectors; the Euclidean distance between these vectors is defined as follows:
$$d\left(R,t_{i}\right)=\sqrt{\sum_{i=1}^{n}{\left(R-t_{i}\right)}^{2}}$$ (2)

c) Arrange the list of all the Euclidean distances in an ascending order
d) k-points are picked out from the training dataset ‘T’
e) Classification task: Classify the test data ‘R’ to class ‘C’, based on the maximum number of feature points near the test data

(or)

Regression task: Calculate the mean of the survival time for these ‘k’ near neighbors. The new mean value indicates the survival time for the test data points **End For**

**Step: iv)** Repeat step iii for all the test data points

## Results

### Comparison of actual and predicted values through error metric – Mean absolute error

Statistical representation is required for estimating the predictive capacity of the model. For this purpose, mean absolute error (MAE) is used ( Botchkarev, 2018). The MAE is the simplest form of calculating the error metric that utilizes the typical magnitude of the residue values. In this method, the residue value is the difference between the actual and predicted values. This value is computed for each input, and the absolute value of these residues is considered such that the positive and negative values will not counteract and nullify. The MAE for each of the ‘k’ scores and the associated input values is calculated as follows:

$$\text{Mean Absolute Error}\;\left(\mathrm{MAE}\right)=\frac{1}{N}\sum_{i=1}^{n}\left(x-x\prime \right)$$ (3)

where *x* is the output value that represents the actual values and *x* is the residue output value that represents the predicted values of the model. The difference between the actual and predicted values given by ( x−x′ ) is the absolute residue value and is calculated for each instance of the input values ranging from *i* = 1 to *n*. Further, ‘ *N*’ indicates the total number of input values.

**Figure 2.**
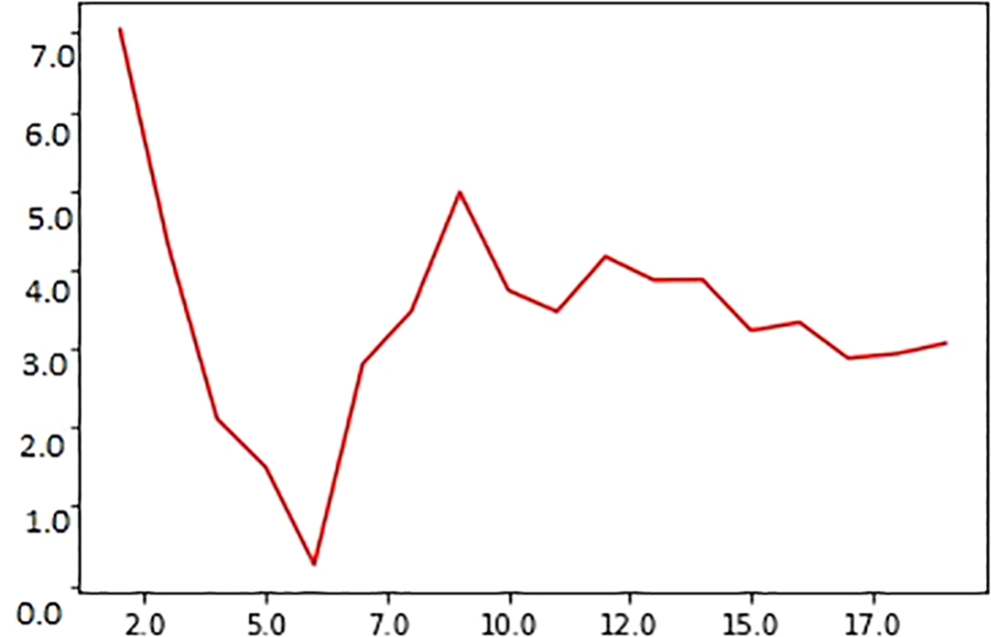
Relationship between ‘k’ value used in the experiment and MAE score for predicting survival time of oral cancer patients. Note that the MAE reaches a minimum (~0.02) score when k = 6; on the contrary, the error value is maximum (~7) when k = 2.

The graphical representation of MAE and the value for ‘K’ are shown in Figure 2. Small MAE values indicate that the difference between the actual and predicted values is low, specifying that the model is good; further, large MAE values suggest that the model is a poor estimator ( Sarker, Iqbal H, 2019). As observed in the figure, the scatter plot of the y-axis represents the coordinates for ‘ *i*’ points of the predicted MAE. The x-axis shows the variations in ‘k’ values. When *x* = x′ , the MAE will show the average vertical distance from the actual and predicted values. The graph between MAE and ‘k’ instances (k = 2 to 20) indicates that the MAE values decrease with an increase in the number of neighbors selected for ‘k’ values. When k = 1, the MAE has a high score, indicating that the error rate is high. The value of ‘k’ was varied from 2 to 19 to determine a suitable ‘k’ value for our experiments. When k = 6, MAE = 0.2, the least in the entire validation curve; thus, for calculating the survival time, ‘k’ was set to 6

### Classification of records to a definite oral cancer stage

**Figure 3.**
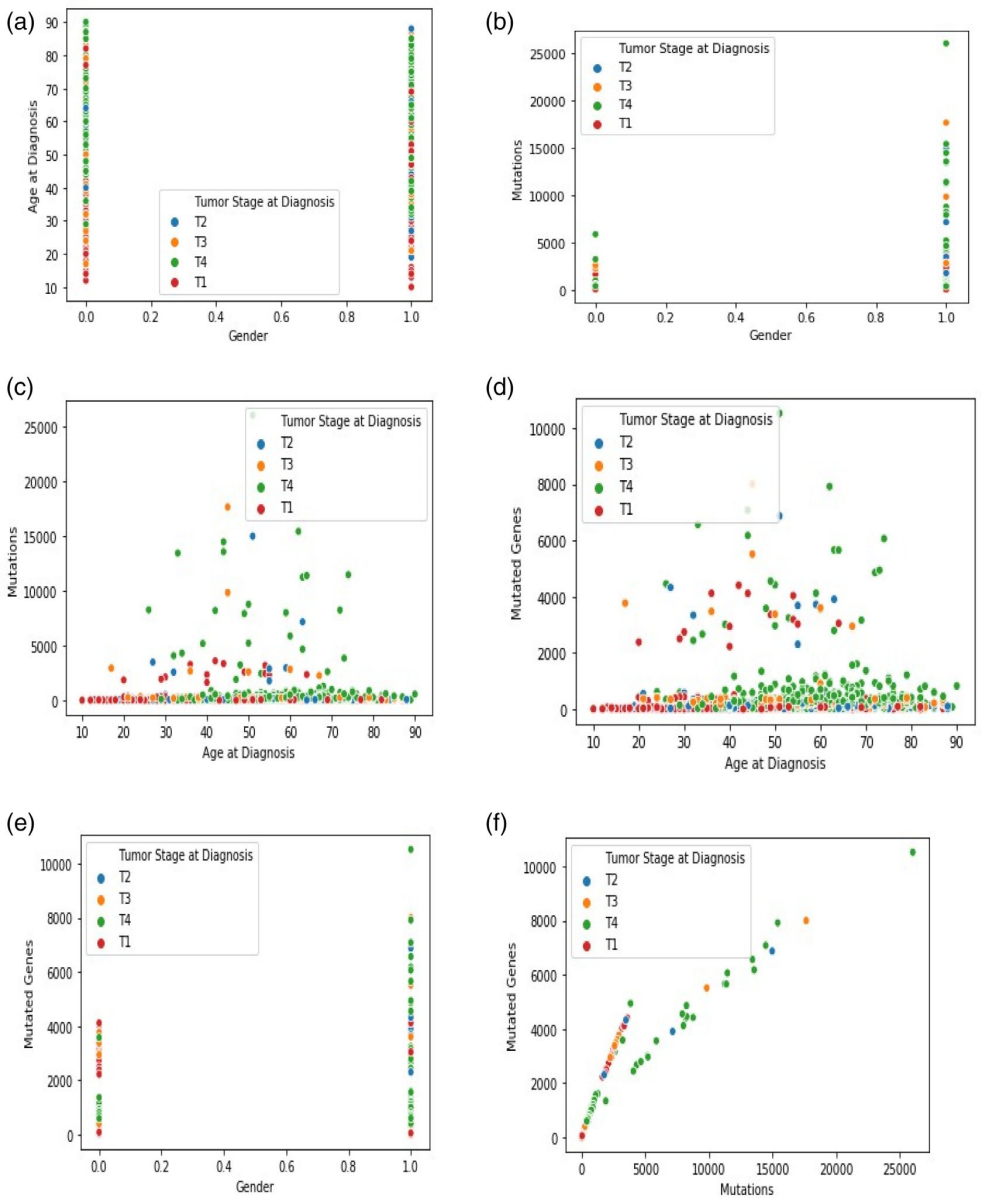
(a). Indication of a gender (0-female, 1-male) and age with the tumor stage. As observed, aged people are more likely to be in the tumor stage-4. (b) Indication of gender and mutations with the tumor stage. As observed, the number of males (1) has a higher number of mutations than females (0). Besides, most of the males are in tumor stage-4. (c) Indication of age at diagnosis and mutations with the tumor stage. As observed, in tumor stage-4, the number of mutations is high and peaks with the increase in a patient’s age. (d) Indication of age at diagnosis and mutated genes with the tumor stage. The number of mutated genes at tumor stage-4 is high, and this pattern is observed in aged patients rather than middle-aged patients. (e) Indication of gender and mutated genes with the tumor stage. As observed, the male (1) experiences more mutated genes than the female (0). The observation also suggests that most of the males are in tumor stage-4. (f) Indication of mutations and mutated genes. As observed, tumor stage-4 has many mutations and mutated genes. Further, for a high number of mutations, the mutated genes are also high in tumor stage-4.

The dataset now contained the predicted survival time values and the previously used parameters and was input into the KNN classifier. Again, to determine the best ‘k,’ the value was varied from 2 to 20. Experimentally, when k = 7, the MAE score attained the most negligible value of 0.1. The cloud of seven neighboring points and the new point is observed in the ‘feature similarity’ space. The test data point is marked with a class label with the highest number of instances in the feature space. If just one example of each class label exists in the feature space, the input data were marked to the closest class. The tumor stages are divided into four variations: T1, T2, T3, and T4. A total of 429 records were efficiently marked into their class belonging using the remaining 1076 data records used as the training data. Figures 3(a) – (f) shows the graphical analysis for different data points. The graph indicates other data points relative to the feature space. Each scatterplot displays the variation of cancer stages and related records with non-identical values at the x- and y-axis. A linear relationship is exhibited between any two variables selected.

## Validation

In this section, the efficiency is measured using accuracy estimation methods, and two main approaches are compared: Hold-out and k-fold cross-validation.

### Error estimation for prediction of survival time using the hold-out cross-validation method

This is the most naïve and straightforward approach of the cross-validation method. The entire dataset is split up only once into training data and test data. The training dataset is chosen randomly, and the fit function is used to train the KNN classifier. The test data, usually 1/3 of the original data, is later predicted and compared with the actual values ( Xu, Yun, 2018). The errors obtained are stored and returned as a mean absolute test error - a risk metric, for evaluating the regression model. The MAE score attained in this method is equal to 9.3, which is relatively high for the model. The high MAE score indicates that the model is performing poorly for the dataset. Interestingly, the cross-validation method consumes minimal time to compute the model’s efficiency, leading to the minimal time complexity of the model. This is potentially due to high variance leading to over-fitting.

### Error estimation for prediction of survival time using the k-fold cross-validation method

To improve over the hold-out method, the data must be split into equally sized observations ( Wong, Tzu Tsung, 2020). The dataset comprised 1505 data entries. For the k-fold error estimation, only 1500 were considered, and five samples were skipped based on selective sampling. A single fold dataset was used for testing, and the remaining k-1 folds were used for training ( Yadav, Sanjay, 2016). According to kfold cross-validation, the entire dataset D _s_ (s = 1, 2, 3, … n) was positioned uniformly. Then dataset D _s_ was divided at k-folds of equal sizes such that each division D _i_ will have ‘k’ number of data points to be evaluated. For experimentation, we used the 10-fold cross-validation method. The dataset contained 1500 records; therefore, F={f _1_,f _2_,f _3_, … .f _1500_}, and each feature f _i_ itself contained attributes such as age, gender, tumor stage, survival time, mutations, and mutated genes. Thus, Eq. (4), explains how the 10fold cross features are selected and provided for the experiment. Since k = 10, the dataset ‘F’ is divided into ten groups such that each group consists of 150 data points.

$$∴10-\text{fold}=\sum_{i=1}^{10}f_{i}\left[1-150\right]$$ (4)

The accuracy is calculated at each fold, and the mean of all the 10-fold accuracies is estimated. The test data are kept isolated at all the iterations at each fold to obtain an unbiased approximate model performance. The test data were never used to fine-tune the model. The entire data set is divided into 10-fold ‘training’ and ‘test’ data. Once the data are divided into 10-folds training and test data, the accuracy is calculated using the standard metric MAE score. The values obtained for the validation error rate are as follows: -0.036, -0.030, 0.014, 0.019, 0.040, 0.035, 0.061, 0.020, 0.028, -0.0001; further, the mean score of the validation error rate is 0.015. The score is perfect as the error rate is negligible and could be rounded to zero. Thus, the k-fold method outperformed the hold-out method in terms of the MAE score when practically applied.

### Validating the classification of patients’ records into specific oral cancer stage through f-measures

Classification accuracy is the number of correct predictions from all the predictions made in the proposed model. With the accuracy measure, the robustness of the model is determined. In most cases, the classifier results are presented in the form of a confusion matrix, which is generally expressed in terms of four measures: true-positive (TP), true-negative (TN), false-positive (FP), and false-negative (FN) ( Lever, Jake, 2016). TP is when the cancer is classified into its correct stage; TN is when a patient’s record is not classified into any wrong stage; FP is when the patient’s record is classified into the wrong stage, e.g., when the patient’s record is ranked as cancer stage-4, but it should have been recognized as cancer stage-3. Finally, FN is when the patients’ record is wrongly classified into a specific stage or sometimes not recognized as a cancer stage. FN leads to disastrous results for cancer datasets as a patient would be detected as not having cancer. Further, no measures would be taken, cancer would aggravate, and the patient will still not be diagnosed until severe symptoms are observed. The subsequent metrics related to accuracy are precision and recall. Precision measures the number of positive predictions over all the positive classes predicted. A recall is a true positive rate that indicates the ratio of all cancer samples that the model accurately predicted. The f-score represents the balancing weight between the recall and precision. Eqs. 5a, 5b, and 5c explain the recall, precision, and Fmeasure, respectively.

$$\text{Recall}\;(R)=\frac{\mathrm{TP}}{\mathrm{TP}+\mathrm{FN}}$$ (5a)

$$\text{Precision}\;(P)=\frac{\mathrm{TP}}{\mathrm{TP}+\mathrm{FN}}$$ (5b)

$$F-\text{Score}=2*\frac{P*R}{P+R}$$ (5c)

**Figure 4.**
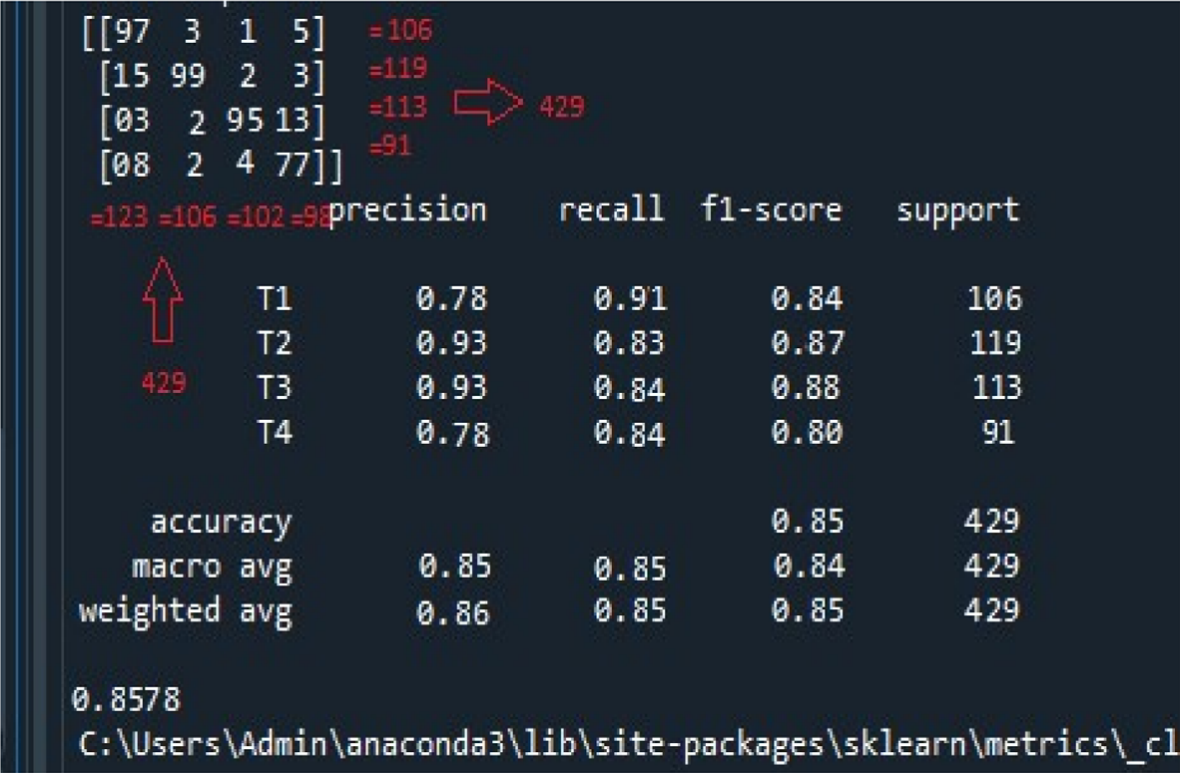
Screenshot of the accuracy metrics obtained by the proposed model. The figure illustrates the following; i) the confusion matrix at the top left panel. There were 429 records used for the classification task. The confusion matrix suggests the recall, f1-score, and iii) accuracy, macro, and weighted average accuracy. Same, both row-wise and column-wise, ii) calculation of precision.

The accuracy achieved for the classification of cancer stages is shown in Figure 4. The model efficiently classified 429 data records into one of the four oral cancer stages.

Initially, the top left panel of Figure 4 indicates the confusion matrix of the four stages of oral cancer obtained with the classifier. As observed, row-wise and column-wise addition of the confusion matrix results in a total of 429 as indicated by support records (106 + 119 + 113 + 91 = 429). Using the formulae, recall, precision, and f-score are calculated for each of the cancer stage. For T1, precision (column-wise representation) = 97 / (97 + 15 + 3 + 8) = 0.78; recall (row-wise representation) = 97 / (97 + 3 + 1 + 5) = 0.91; and f-score = (2 * 0.78 * 0.91) / (0.78 + 0.91) = 0.84. For T2, precision = 99 / (3 + 99 + 2 + 2) = 0.93; recall = 99/ (15 + 99 + 2 + 3) = 0.83; and f-score = (2 * 0.93 * 0.83) / (0.93 + 0.83) = 0.87. For T3, precision = 95 / (1 + 2 + 95 + 4) = 0.93; recall = 95/ (3 + 2 + 95 + 13) = 0.84; and f-score = (2 * 0.93 * 0.84) / (0.93 + 0.84) = 0.88. Finally, for T4, precision = 77 / (5 + 3 + 13 + 77) = 0.78; recall = 77/ (8 + 2 + 4 + 77) = 0.85; and f-score = (2 * 0.78 * 0.84) / (78 * 0.84) = 0.80. The overall accuracy is equal to the number of correctly classified samples (97 + 99 + 95 + 77) divided by total number of samples (429) = 0.85. To verify the overall f-score of the classifier, macro and weighted average arithmetic mean are considered. The macro-averaged score is the arithmetic mean of all the recall, precision, and f1-scores obtained thus far. Therefore, the macro-average score for precision = (0.78 + 0.93 + 0.93 + 0.78) / 4 = 0.85; recall = (0.91 + 0.83 + 0.84 + 0.84) / 4 = 0.85; and f1-score = (0.84 + 0.87 + 0.88 + 0.80) / 4 = 0.84. The weighted average score is calculated as the product of the weighted sum of each of the recall, precision, and f1-scores and individual support records over the total number of support records for each cancer stage (429). The weighted average for precision = (0.78 * 106 + 0.93 * 119 + 0.93. * 113 + 0.78 * 91) / 429 = 0.86; recall = (0.91 * 106 + 0.83 * 119 + 0.84 * 113 + 0.84 * 91) / 429 = 0.85; and f1-score = (0.84 * 106 + 0.87 * 119 + 0.88 * 113 + 0.80 * 91) /429 = 0.85. Finally, the accuracy is calculated by looking at all the samples at once and identifying the correctly predicted cancer stages (TP) present in the mid-diagonal of the confusion matrix over the sum of correctly predicted samples (TP) and falsely predicted samples (FP). Therefore, the overall accuracy obtained by the proposed classifier is calculated as follows: (97 + 99 + 95 + 77) / [(97 + 99 + 95 + 77) + (3 + 1 + 5 + 15 + 2 + 3 + 3 + 2 + 13 + 8 + 2 + 4)] = 0.8578. Thus, the proposed classifier for efficiently segregating oral cancer data records into various stages in a multi-class classification is 85.7% accurate, and the same demonstrated in the experimental results.

## Discussion

The present study was conducted to review the association between the clinical factors and patients’ survival time. The research could considerably influence the treatment cycle and subsequent intervention in the prognosis procedure. The correlation between cancer patients’ mutation and mortality rate indicates that older patients with several mutations had a short survival time. In contrast, even with an average number of mutations, say 2000-4000, the survival time was long for a young patient. The predicted survival time of patients at their early cancer stage displayed a longer survival time of 5 – 7 years, followed by metastatic cancers. Also, patients with a short survival time were classified into cancer stage-4. Thus, the study highlights the following: i) more males are diagnosed with oral cancer than females; ii) the number of mutations increases with the patients’ age; iii) when the mutation number is high, the number of genes that are mutated is also high; and iv) tumor stage-4 has more number of mutations and mutated genes and entails more of the older population than the younger people. This indicates that the treatment selection and diagnosis are influenced by these fundamental factors that most clinical studies overlook; as with the regression technique, the present study models the effect of multivariate data points used as the input variables for the execution. The dataset may be scaled up to include many datasets with the specific tumor stage classification to understand efficacy better. Factors such as age and mutations directly impact the survival rate of the patient.

The present study has some limitations. As pointed out earlier, the corpus size in ML-based research contributes to the system’s robustness and accuracy. When the corpus size is relatively small, the model suffers from overfitting leading to misclassification of cancer stage and incorrect survival time prediction. Along with this, feature selection is a crucial ingredient for integrity. A simple wrong entry one row down by an operator may lead to inconsistent data, eventually piling up many errors in the later evaluation stage. While selecting essential features from the dataset, factors such as measurability, pathological assessments, and relevance must be considered, which the present study failed to grab. The future directions of the research work are: i) apply ML algorithms to analyze both clinical and genomic factors to study which mutations are predominantly contributing to causing cancer, ii) ML-based pathological model, such that, when the data is input sitting at home or clinic, it predicts the cancer progress and gives a report on survival time, treatments that are likely to be followed and drugs based on the genomic make-up of the patient, iii) develop pre-clinical ML model to control cancer development through early diagnosis, deliver clinical trials with higher accuracy, iv) the gene-specific reactions should be investigated and their role in inducing the mutations in the protein structure and the corresponding relevance on the survival time. This would enable clinicians to adopt effective, targeted therapy options.

## Conclusion

Over the past few decades, the advent of ML-based algorithms for cancer treatment has paved the way for better prognosis procedures. However, the prediction of survivability by analyzing the clinical factors present in EMRs is often neglected. In this study, KNN, a supervised machine learning approach, was applied to precisely estimate the survival time of an oral cancer patient. The study also classifies the dataset into a specific cancer stage. With the increasing number of deaths due to oral cancer, the purview of such a study is necessary. The fundamentals of survival time analysis were explored using the data available, and the limitations owing to a poor diagnosis were discussed. A whole new treatment procedure could be implemented if the survival time is known. In precision medicine, identifying the key proteins and mutated genes is essential for devising the treatment strategies. Most importantly, the proposed approach illustrates the use of digital data collected by the health care system consistently but underexploited by clinicians. The study demonstrates the accuracy measures using cross-validation and f- scores.

## Data availability

For ease of use of the proposed methodologies, the entire code and all the datasets with relevant results have been deposited at the GitHub repository ( https://github.com/RashmiSKarthik/Machine). The data has also been deposited on Zenodo (10.5281/zenodo.5819317). The code could be scaled up to other cancer datasets, and the model works efficiently on any Python platform. Any copyrighted material of this research can be reproduced with appropriate work citations.

The repository is publicly available. Any queries and concerns related to code and implementation may be directed to the corresponding author of this manuscript.

## Competing interests

The author(s) declare that there are no potential conflicts of interest concerning this current research, authorship, and/or publication of this work
