# Supplementary material for: K-nearest-neighbor algorithm to predict the survival time and classification of various stages of oral cancer: a machine learning approach
Source: F1000Res. 2023 Nov 16;11:70. Originally published 2022 Jan 20. [Version 2] doi: 10.12688/f1000research.75469.2 (PMC10690040; doi:10.12688/f1000research.75469.2)
Supplement: Peer review history

# Reviewer report: Reviewer response for version 2

**DOI:** 10.5256/f1000research.156859.r223448

**Published:** 2023-11-30

**Authors:** Harshad Hegde

**Affiliations:** 1 Lawrence Berkeley National Laboratory, Berkeley, CA, USA

I believe the quality of the paper has improved significantly and I commend the author for addressing my previous review.

Is the work clearly and accurately presented and does it cite the current literature?

Yes

If applicable, is the statistical analysis and its interpretation appropriate?

Yes

Are all the source data underlying the results available to ensure full reproducibility?

Yes

Is the study design appropriate and is the work technically sound?

Yes

Are the conclusions drawn adequately supported by the results?

Yes

Are sufficient details of methods and analysis provided to allow replication by others?

Yes

Reviewer Expertise:

I have been a bioinformatics researcher for almost a decade.

I confirm that I have read this submission and believe that I have an appropriate level of expertise to confirm that it is of an acceptable scientific standard.

# Reviewer report: Reviewer response for version 1

**DOI:** 10.5256/f1000research.79347.r201257

**Published:** 2023-08-30

**Authors:** Harshad Hegde

**Affiliations:** 1 Lawrence Berkeley National Laboratory, Berkeley, CA, USA

1. "According to the GLOBOCAN 2008 estimate " - A more recent statistic would be helpful (from WHO, maybe?). 
2. I don't see the full-form for MAE which should be at the first place it was used. I see it in the "Results" section which is after reading the abbreviation at least 3 times prior.
3. So as far as I understand, only 4 features were used (age, gender, mutations, and mutated genes)? " The remaining fields are dropped" - Was there a reason? Please explain. Was the decision on these features based on specific literature or arbitrary?
4. Explanation for the data normalization stage in detail would be very helpful for recreating the study for future research. For e.g. " tumor stage by alphanumeric values" - how was this information "normalized" to make it classifier friendly? Did you one-hot encode it or use the features as boolean? The paper does not mention this. A detailed explanation about the data ETL (extract-transform-load) right from the data ingest stage up to the point of classifier ready input data is crucial.
  "multiple fields had null or no weights" - how did you tackle the issue? Were there steps taken to handle missing data (e.g. data imputation etc.)?  Show examples of data in the form of small tables to make these things clear for readers.
5. Were other classifiers besides kNN considered? If not, why? If yes, results from those for comparison would be helpful.
  "Further, they used various classification algorithms such as Naïve Bayes, KNN, support vector machine (SVM), and decision trees to yield specific gene mutations efficiently" -Other studies have clearly used other classifiers. The paper would be stronger if you ran your data against the ones above and/or others (like random forests, MLP etc.) that are appropriate for your data volume and reporting comparisons.
6. The results could be better presented in the form of a neat table or a figure so that it is easily legible.

Is the work clearly and accurately presented and does it cite the current literature?

Yes

If applicable, is the statistical analysis and its interpretation appropriate?

Yes

Are all the source data underlying the results available to ensure full reproducibility?

Yes

Is the study design appropriate and is the work technically sound?

Yes

Are the conclusions drawn adequately supported by the results?

Yes

Are sufficient details of methods and analysis provided to allow replication by others?

Yes

Reviewer Expertise:

I have been a bioinformatics researcher for almost a decade.

I confirm that I have read this submission and believe that I have an appropriate level of expertise to confirm that it is of an acceptable scientific standard, however I have significant reservations, as outlined above.

# Response

**Published:** 2023-09-29

**Authors:** Rashmi Siddalingappa

**Affiliations:** Indian Institute of Science, India

The authors of this paper thank the reviewer for the comments and below are our responses for the same: 

 Answer to question 1: According to WHO, there were about 10 million deaths worldwide due to oral cancer in 2020 (Ferlay et al., 2020)

 Answer to question 2: Earlier, the comparison analysis section has given MAE full form. however, the authors now have included the MAE full form at the beginning of the paper, where the term gets introduced first

 Answer to questions 3 and 4: The data cleaning section has been completely rewritten, which now addresses all the concerns that were raised, such as ETL, Handling Missing Data, and Normalization.

 Answer to question 5: Yes, we agree, and that is why a comparison study was done w.r.t to the KNN classifiers with other traditional ML classifiers such as SVM and RF, and the results of the same are discussed in Table 1.

 Answer to question 6: The results of our work now are pictorially represented in the form of a figure/plot, as shown in Figure 5. Accordingly, some changes have been made in this section to talk about the performance metrics with better clarity.

# Reviewer report: Reviewer response for version 1

**DOI:** 10.5256/f1000research.79347.r121749

**Published:** 2022-02-03

**Authors:** Shivanand Sharanappa Gornale

**Affiliations:** 1 Department of Computer Science, Rani Channamma University, Belagavi, Karnataka, India

The paper has significant merits about the implications of Machine Learning (ML) techniques in predicting the survival of oral cancer patients and further performing the classification tasks on the medical records. The introduction and positioning of the work in the general context of oral cancer and ML is done well, with appropriate metrics.

**Strengths**

- This is a well-written paper with detailed information about the impact of cancer on the current trend.
- The authors have precisely explained how cancer succumbs people and what the prime causes are.
- Further, the authors discuss how ML can impact predicting the survival time of cancer. The authors point out the merits of this prediction to clinicians, patients, and researchers
- The authors have performed the classification of the predicted data into various oral cancer stages
- It is the first time introducing KNN as classification and regression tasks to predict the survival time based on the number of mutations and age.
- The ‘k’ component was measured experimentally in the research, which would evade any hypothetical argument on why a particular value of ‘k’ is fixed. The authors have shown varying results for different ‘k’ values starting from 2 to 20.
- The story is straightforward, and the data underlines the argumentation clearly.
- The conclusions are persuasive and very important for oral cancer prognosis procedures. This work would inspire more research concentrating on the survival prediction analysis, thereby improving the treatment modification for patients’ right from the diagnosis stage.
- References citations are appropriate.

**Weaknesses**

  

- The authors may apply these strategies using advanced ML techniques in the future work of the research study
- The methodology is generally acceptable. Some general data on geographically-separated populations may be done in the future to fully understand the potentiality/impact of the work for survival prediction
- It would be nice to have some discussion on the uncertainties/requirements of the ML involvement in clinical studies like this one.The paper has significant merits about the implications of ML techniques in predicting the survival of oral cancer patients and further performing the classification tasks on the medical records. The introduction and positioning of the work in the general context of oral cancer and ML is done well, with appropriate metrics.

Is the work clearly and accurately presented and does it cite the current literature?

Yes

If applicable, is the statistical analysis and its interpretation appropriate?

Yes

Are all the source data underlying the results available to ensure full reproducibility?

Yes

Is the study design appropriate and is the work technically sound?

Yes

Are the conclusions drawn adequately supported by the results?

Yes

Are sufficient details of methods and analysis provided to allow replication by others?

Yes

Reviewer Expertise:

Digital Image Processing and Pattern Recognition, Biometric Data Analysis, Natural Language Processing

I confirm that I have read this submission and believe that I have an appropriate level of expertise to confirm that it is of an acceptable scientific standard.
